# Analysis of the Comorbid Course of Chronic Obstructive Pulmonary Disease

**DOI:** 10.3390/jpm13071179

**Published:** 2023-07-24

**Authors:** Stanislav Kotlyarov

**Affiliations:** Department of Nursing, Ryazan State Medical University, 390026 Ryazan, Russia; skmr1@yandex.ru

**Keywords:** COPD, ASCVD, atherosclerosis, comorbidity, survival

## Abstract

(1) Background. Chronic obstructive pulmonary disease (COPD) has a heterogeneous natural history, manifested both in the variability of clinical features and in association with various comorbid pathologies. Atherosclerotic cardiovascular disease (ASCVD) is of great clinical importance and contributes significantly to the natural history and prognosis of COPD. The present study aimed to evaluate the nature of the comorbid course of COPD during a 15-year follow-up. (2) Methods: A total of 170 male COPD patients were included in this study. Spirometry values, symptom severity, presence of risk factors, and comorbidities were considered. Prognostic factors were evaluated using the Kaplan–Meier method. (3) Results: ASCVD was the most common comorbidity and the main cause of death in patients with COPD. Patients with comorbid COPD and ASCVD had more severe dyspnea, higher frequency of COPD exacerbations, and worse survival than patients without ASCVD (*p* < 0.01). Among patients with COPD, the risk of death from ASCVD was higher in those older than 60 years (OR 3.23, 95% CI [1.72, 6.07]), those with rapidly declining FEV1 (OR 4.35, 95% CI [2.28, 8.30]), those with more than two exacerbations per year (OR 3.21, 95% CI [1.71, 6.11]), and those with a pack year index greater than 30 (OR 2.75, 95% CI [1.38, 5.51]. High Charlson comorbidity index scores in patients with COPD were associated with a more severe disease course, including severity of dyspnea, frequency of exacerbations, and multivariate index scores. A high Charlson comorbidity index score was an adverse prognostic factor. (4) Conclusions: ASCVD influences the course of the disease and is a major cause of mortality in COPD patients.

## 1. Introduction

Chronic obstructive pulmonary disease (COPD) is an important medical problem associated with negative trends in the prevalence of the disease, as well as its contribution to the structure of claims for medical care and temporary and permanent disability [1,2]. The disease is among the leading causes of mortality and occupies third place in this rating [2,3]. In addition, COPD has a heterogeneous course characterized by variability in clinical manifestations [4]. The heterogeneous course of COPD is the basis for attempts to identify phenotypes of the disease, which could be taken into account when choosing tactics for patient management or estimating prognosis [5]. An important manifestation of the clinical heterogeneity of COPD is the presence of comorbidities, which may modify the course of the disease and affect its prognosis [6,7]. Atherosclerotic cardiovascular disease (ASCVD) is the most important comorbidity of COPD [7,8]. Atherosclerotic lesions of three key vascular basins that cause coronary artery disease (CAD), cerebral ischemia, and peripheral arterial disease (PAD) have the greatest clinical significance [9,10]. The close relationship between COPD and CAD, and carotid and peripheral artery lesions is well known [7,11]. The importance of the problem of the combined course of COPD and ASCVD is underscored by the fact that both diseases are often not diagnosed in time, since, for example, PAD may be asymptomatic for a long time and only becomes apparent in late stages with the appearance of intermittent claudication, pain in the lower limbs at rest, as well as non-healing wounds, ulcers, and gangrene [7,12].

Evidence suggests that the bronchitis phenotype of COPD is more commonly associated with atherosclerosis [13]. It was also suggested that COPD may contribute to diffuse atherosclerotic vascular lesions, characterized by worse clinical outcomes [14]. This may be due to the presence of common links in the pathogenesis of COPD and atherosclerosis, including the roles of oxidative stress and smoking-induced systemic inflammation [15,16,17]. COPD exacerbations contribute to the severity of systemic inflammation, which is an important link between COPD and comorbid diseases.

Although many studies analyzed the significance of comorbid diseases in the course of COPD, many clinical features of comorbidity remain debatable. However, there is no doubt that comorbid diseases in COPD can be considered a part of its extrapulmonary clinical heterogeneity. Several tools were proposed to assess comorbidities in COPD, including the Charlson comorbidity index (CCI), which was shown to be effective in assessing the course and prognosis of COPD [18,19,20]. Clinical evidence suggests that the comorbid course of COPD and ASCVD can modulate the clinical picture. The disease leads to limitations in physical activity and the occurrence of symptoms such as dyspnea. ASCVD is also a leading cause of death in COPD patients.

Thus, COPD is a clinically heterogeneous disease characterized by differences in clinical and functional characteristics, such as the severity of symptoms, frequency of exacerbations, and structure of comorbid pathology. These differences play an important role in influencing the disease course and prognosis. A better understanding of these characteristics would improve the quality of patient management in clinical practice. Taking these and other data into account, the aim of the present study was to assess the nature of the comorbidity course of COPD in a fifteen-year follow-up.

## 2. Materials and Methods

### 2.1. Study Design

**Figure 1:**
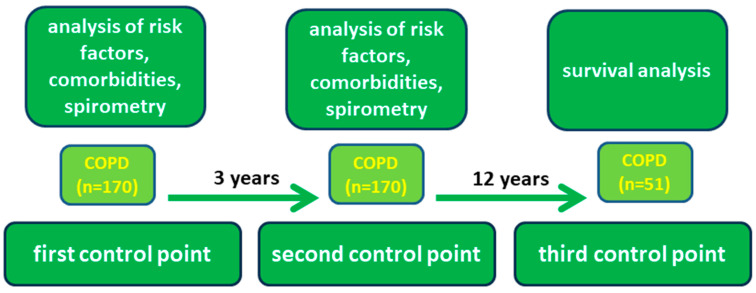
Study design.

The study was conducted over a 15-year period and patients were examined at baseline (first control point, 2007) and 3 years later (second control point, 2010) to assess disease progression. Survival was then followed for the next 12 years and factors influencing prognosis were analyzed (Figure 1).

Risk factors, the presence of chronic respiratory symptoms, and an examination of respiratory function, including forced expiratory volume in one second (FEV1), were analyzed at the first and second control points. The results of post-bronchodilator spirometry were considered. All patients underwent spirometry twice: the first time at baseline and three years later at the second control point to assess the rate of FEV1 decline. A relative decline in FEV1 of more than 100 mL/year was considered a rapid decline.

Smoking status and working in dusty air pollution conditions were analyzed as risk factors. Smoking history was assessed using the pack-years index. Work in conditions of dust air pollution was determined in accordance with the lists of harmful production factors at workplaces, established by the results of a special assessment of working conditions.

Dyspnea severity was assessed using the modified Medical Research Council (mMRC) dyspnea scale. COPD exacerbation was defined as worsening of ≥2 major symptoms (dyspnea, purulent sputum, or sputum volume) or worsening of any one major symptom and a minor symptom (sore throat, nasal discharge, fever without other causes, cough, or wheezing) for two or more consecutive days [21]. Exacerbations were assessed according to patient questionnaires and reasons for seeking medical care. To objectify the data, the mean frequency of exacerbations per year was calculated considering the three-year data between the first and second control points. Comorbid conditions were assessed using the CCI. CCI was calculated using age and presence of somatic diseases. In addition, multidimensional indices, including BODE, BODEX, eBODE, and CODEX, were calculated. The BODE index considers body mass index (B), degree of airway obstruction (O), severity of dyspnea according to the mMRC scale (D), and exercise intolerance (E), as measured by the six-minute walk distance (6 MWD). The BODEX index also considers the frequency of COPD exacerbations (EX) [22]. The e-BODE index takes into account the body mass index (B), degree of airflow obstruction (O), mMRC dyspnea severity (D), exercise intolerance (E) as assessed by the 6 MWD, and exacerbation frequency (EX). The CODEX index includes comorbidity data based on the CCI (C), degree of airflow obstruction (O), dyspnea severity on the mMRC scale (D), and exacerbation frequency (EX). The current study accounted for ASCVD, which includes clinically manifested forms of coronary, cerebral atherosclerosis, and lower extremity arterial atherosclerosis, including coronary heart disease, peripheral arterial disease, and cerebrovascular disorders.

The third control point included medical record data on medical treatment and hospitalization. Comorbid conditions were identified by examining and analyzing medical records. For deceased patients, the cause of death was analyzed according to the medical records.

### 2.2. Study Participants

This study included 170 male COPD patients living in the Ryazan region. Patients were selected for the study using a two-stage method. The first part of the sample consisted of patients with COPD who visited eight primary health care facilities during a given period. The second part of the sample consisted of people who lived in specific settlements and were included in the study through door-to-door visits, selected by random numbering from the population list after confirmation of the COPD diagnosis. This made the sample larger and more representative. In the preliminary phase, patients completed a questionnaire, as well as underwent spirometry and clinical examination to confirm the diagnosis.

The inclusion criteria were age > 35 years, voluntary informed consent to participate in the study, and a diagnosis of COPD confirmed by spirometry according to the Global Initiative for Chronic Obstructive Lung Disease (GOLD) criteria. Patients with comorbidities that were too severe to allow for long-term follow-up were excluded from the study. Patients with known cancer at any site, recent myocardial infarction and acute stroke (less than 6 months), severe congestive heart failure, HIV infection, and other immunodeficiencies, and those unable to understand and comply with the study protocol were also excluded. According to GOLD criteria, 53 patients (31.17%) with stage I COPD (mild), 104 (61.23%) with stage II COPD (moderate), and 13 (7.6%) with stage III COPD (severe) were included in the study.

All patients enrolled in the study were prescribed appropriate pharmacotherapy according to the severity of COPD and the presence of comorbidities, taking into account the requirements of GOLD and national clinical guidelines and treatment protocols, as well as individual clinical indications.

### 2.3. Statistical Analysis of Data

Statistical processing and visualization of the results were performed using RStudio (v.4.0.2) and http://www.bioinformatics.com.cn (accessed on 25 May 2023). Data are presented as mean and its 95% confidence interval (CI). Categorical data were compared using the chi-squared test, and continuous variables were compared using Student’s *t*-test or Mann–Whitney U test, analysis of variance (ANOVA), or Kruskal–Wallis ANOVA after evaluation of criteria for use of parametric tests. Survival analysis was calculated using the Kaplan–Meier method. Statistical significance of curve differences was assessed using the log-rank test. Differences were considered statistically significant at *p* < 0.05. A logistic regression model was used to calculate odds ratios (OR).

### 2.4. Ethical Approval

The protocol of this study was approved by the Ethical Committee of the Ryazan State Medical University (Protocol No. 2, 12 November 2007).

## 3. Results

### 3.1. Clinical Characteristics of Patients

The demographic and baseline clinical characteristics of the patients included in the study are presented in Table 1. The cohort consisted of men at a mean age of 60.02, 95% CI [58.68, 61.34] years. All patients were smokers at the time of study inclusion. The mean smoking history was 37.72, 95% CI [36.41, 39.03] pack-years.

The most common comorbidities in patients were cardiovascular diseases, such as arterial hypertension (64.7%) and CAD (54.11%), with a history of myocardial infarction (MI) in 6.47% of the patients. PAD was present in 8.82% of the patients (Table 2). Type 2 diabetes mellitus was present in 18 (10.58%) patients, and diseases of the genitourinary system (chronic pyelonephritis, urolithiasis, and chronic kidney disease) were present in 19 (11.17%) patients. 

The risk of ASCVD in patients with COPD increased with age (OR 1.18, 95% CI [1.12, 1.24]), especially after the age of 60 years (OR 10.60, 95% CI [5.20, 21.61]). The risk of ASCVD increased with an increase in the pack-year index (OR 1.08, 95% CI [1.03, 1.12]), with a frequency of COPD exacerbations more than twice per year (OR 3.02, 95% CI [1.61, 5.65]). Meanwhile, the risk of PAD also increased with age (OR 1.08, 95% CI [1.01, 1.16]), especially in those older than 60 years (OR 4.15, 95% CI [1.13, 15.31]), with increasing pack-year index (OR 1.10, 95% CI [1.03, 1.18]), and with the frequency of COPD exacerbations of more than twice a year (OR 15.32, 95% CI [1.96, 119.41]).

### 3.2. Survival Analysis and Evaluation of Prognostic Factors

The analysis revealed different clinical and functional characteristics of the course of COPD and their influence on prognosis. The fifteen-year survival rate was 30% and 119 patients died. The mean life expectancy of those who died was 70.48 (95% CI [69.23, 71.73]) years. Analysis of the cause of death showed that major adverse cardiovascular and cerebrovascular events were the leading causes of death in patients with COPD, including cerebrovascular disease (45 patients, 37.81%), coronary artery disease (36 patients, 30.25%), and peripheral artery disease complicated by gangrene (2 patients, 1.68%). Other significant causes of death were cancer (16 patients, 13.44%) and COPD (12 patients, 10.08%). Other causes, including trauma and cirrhosis, accounted for 8 patients (6.72%).

**Figure 2:**
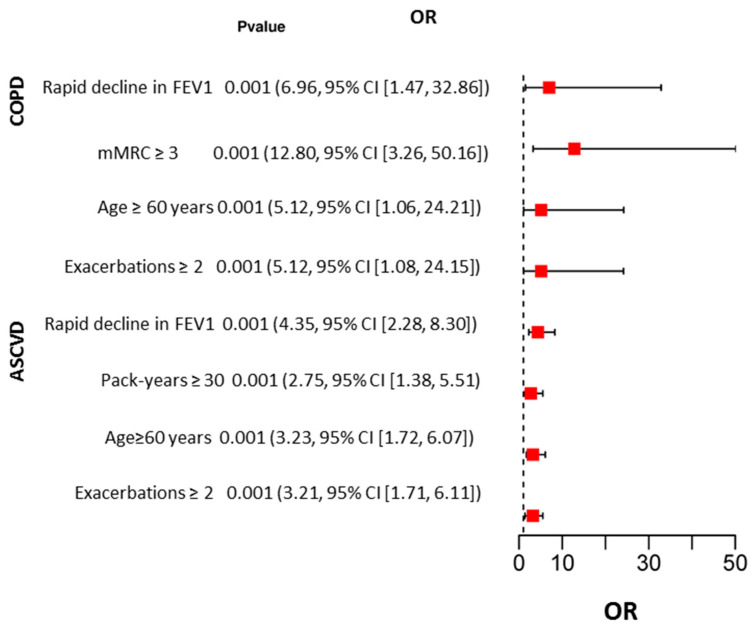
Results of univariate logistic regression analysis of factors influencing COPD and ASCVD deaths.

Logistic regression analysis showed that the risk of death from COPD was higher in patients with rapidly declining FEV1 (OR 6.96, 95% CI [1.47, 32.86]), frequency of COPD exacerbations more than twice per year (OR 5.12, 95% CI [1.08, 24.15]), dyspnea score greater than 3 on the mMRC scale (OR 12.80, 95% CI [3.26, 50.16]), and age older than 60 years (OR 5.12, 95% CI [1.06, 24.21]). The risk of death due to ASCVD was higher in patients older than 60 years (OR 3.23, 95% CI [1.72, 6.07]), those with rapidly declining FEV1 (OR 4.35, 95% CI [2.28, 8.30]), those with a frequency of COPD exacerbations greater than twice per year (OR 3.21, 95% CI [1.71, 6.11]), and those with a pack-year index greater than 30 (OR 2.75, 95% CI [1.38, 5.51]) (Figure 2).

The risk of rapid decline in FEV1 was significantly increased with increasing COPD severity (HR 3.75, 95% CI [1.97, 7.15], increasing CCI (HR 1.39, 95% CI [1.14, 1.70], frequency of COPD exacerbations more than two per year (HR 3.87, 95% CI [1.97, 7.57], and presence of comorbid ASCVD (HR 2.85, 95% CI [1.47, 5.51]. An evaluation of the rate of FEV1 decline showed that a rapid decline in FEV1 (≥100 mL per year) was a negative prognostic factor (HR 5.62, 95% CI [3.66, 8.63], *p* < 0.001).

### 3.3. Analysis of Comorbidities in COPD

**Figure 3:**
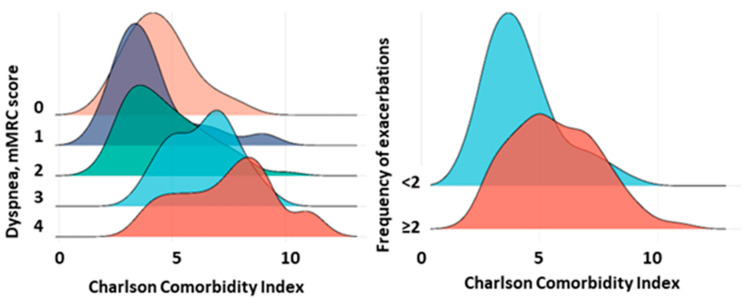
CCI in patients with different severity of dyspnea and frequency of COPD exacerbations. Note: The figures show CCI values for different severity of mMRC dyspnea and different frequency of exacerbations (<2 and ≥2 per year).

Analysis of the CCI showed that the values had a moderate correlation with the number of years lived from the first reference point (r = −0.43, 95% CI [−0.54, −0.29], *p* < 0.0001), the severity of dyspnea scores (r = 0.42, 95% CI [0.29, 0.54], *p* < 0.0001), and the dynamics of decline in FEV1 (r = 0.40, 95% CI [0.26, 0.52], *p* < 0.0001). However, the CCI showed a weak correlation with COPD stage (r = 0.25, 95% CI [0.11, 0.39], *p* < 0.0001). An increase in dyspnea severity corresponded to an increase in the CCI (Figure 1). In addition, patients with two or more COPD exacerbations per year had higher CCI scores (*p* < 0.001) (Figure 3).

**Figure 4:**
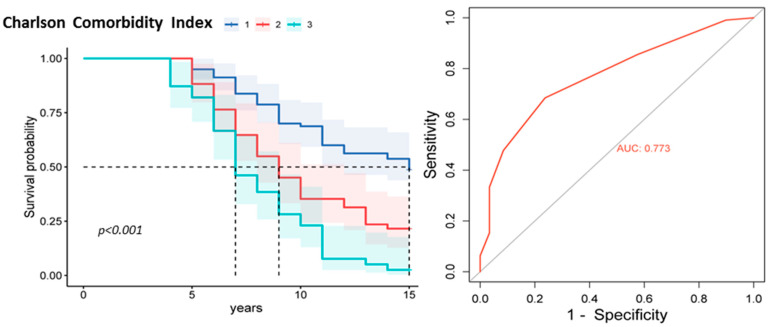
Kaplan–Meier survival curve plot and ROC curve for the CCI. Note: CCI subgroup 1 is a score of 1 to 4, subgroup 2 is a score of 5 to 6, and subgroup 3 is a score of 7 or higher.

It was also found that increased CCI values were associated with poor prognosis (Figure 4). The Kaplan–Meier survival curve showed that patients with high CCI scores had significantly worse survival than those with lower index scores (*p* < 0.001). ROC analysis confirmed the significance of the CCI as a predictor of adverse outcomes. The area under the ROC curve (AUC) was 0.77, 95% CI [0.69, 0.83], indicating good prognostic significance.

CCI values showed statistically significant differences between the survivor and deceased groups during the fifteen-year follow-up period. Among the patients who survived the follow-up period, the CCI at the second control point was 3.76, 95% CI [3.42, 4.11] points, while among those who died, it was 5.5, 95% CI [5.17, 5.84] points (*p* < 0.001). In a logistic regression model comparing patients with the lowest CCI scores (1–4 points) and those with high CCI scores (greater than 7 points) at the second control point, the odds ratio (OR) for the risk of death from ASCVD was 8.44, 95% CI [2.64, 27.00], and for the risk of death from COPD was 18.40, 95% CI [2.04, 165.36].

In the present study, ASCVD was found to have a significant effect on the course of COPD (Table 3). Patients with comorbid ASCVD had more severe dyspnea on the mMRC scale and longer duration of chronic respiratory symptoms, such as cough and sputum production. The 5-year, 10-year, and 15-year survival rates were lower in the group of patients with COPD combined with ASCVD (Table 3). 

In a logistic regression model comparing patients in the subgroup with the lowest FEV1 (% pred) (≤35%) with patients in the subgroup with the highest FEV1 (≥80) at the second control point, the odds ratio (OR) for the risk of ASCVD was 12.00 (95% CI, 1.48–96.68). Thus, low FEV1 was associated with the presence of ASCVD.

**Figure 5:**
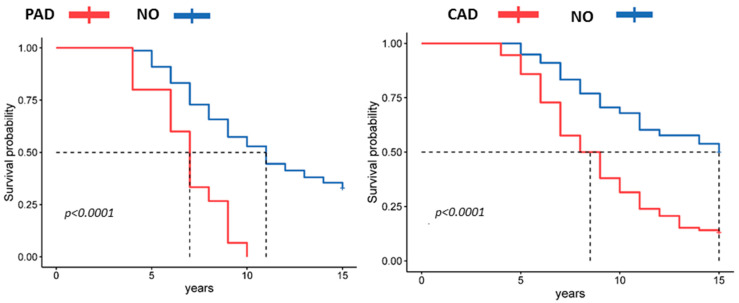
Kaplan–Meier curve plots of survival according to the presence of comorbid PAD and CAD.

Analysis of the concomitant ASCVDs that the patients had at the time of study initiation showed that the presence of CAD and PAD in patients with COPD was a predictor of adverse prognosis (Figure 5). The Kaplan–Meier survival curve showed that patients with comorbid clinically significant PAD and CAD at baseline had a significantly lower survival rate than patients without these comorbidities (*p* < 0.001).

The multidimensional indices BODE, eBODE, BODEX, CODEX, and the Charlson comorbidity index assessed at the second control point showed statistically significant differences between the two groups (Table 4). These data suggest that COPD combined with ASCVD is characterized by a more severe course and greater comorbidity.

In a logistic regression model comparing patients in the lowest CODEX index quartile (0–2 points) with those in the highest CODEX index quartile (7–10 points) at the second control point, the odds ratio (OR) was 7.61, 95% CI [1.88, 30.81] for the 5-year risk of all-cause death, (OR) 7.31, 95% CI [2.39, 22.33] for the 5-year risk of death from ASCVD, and (OR) 15.69, 95% CI [2.84, 86.48] for the 5-year risk of death from COPD. Thus, the CODEX index, which considers comorbidities, is of clinical interest in assessing the course and prognosis of COPD.

To evaluate the influence of different factors and their combinations on long-term survival of COPD patients, multifactorial analysis of long-term (15-year) survival was performed with the Cox regression model (proportional hazards model) using the method of sequential exclusion of variables. The relative risks of the influence of each factor on the probability of COPD and ASCVD mortality in the long-term period were calculated. Predictors were analyzed for patients with COPD-related cause of death and for patients who died from ASCVD (Table 5).

Thus, the results of the optimized Cox regression model show that the significant predictors of death in patients with COPD in long-term follow-up were: frequency of exacerbations more than two per year, rapid decline in FEV1, smoking history more than 30 pack-years, and presence of ASCVD in the patient’s history

Thus, analysis of COPD progression over a fifteen-year period showed that comorbid ASCVD influences COPD progression and prognosis and should be considered when monitoring COPD.

## 4. Discussion

In this study, we evaluated the comorbidity course of COPD over a fifteen-year follow-up period. A total of 170 men diagnosed with COPD according to the GOLD criteria were included in this study. Patients were examined at baseline, three years after study initiation, and then followed up for 12 years. The study included an analysis of medical history and clinical data, including exacerbation frequency, respiratory symptom severity, lung function, and presence of comorbidities.

We showed that ASCVD is the leading cause of death in patients with COPD. Moreover, a history of ASCVD is associated with poor prognosis. Patients with COPD and ASCVD had a more severe course, characterized by a greater severity of dyspnea and higher values of multidimensional prognostic indices. These results also suggest that the CCI is an important clinical indicator that can be used to assess the prognosis of COPD. An increase in the CCI score is associated with a worse prognosis.

COPD is a clinically heterogeneous respiratory disease characterized by chronic airway inflammation, leading to airway obstruction and chronic respiratory symptoms [23]. Inflammation in COPD involves both the local and systemic components. Systemic inflammation is an important cause of the extrapulmonary clinical heterogeneity of COPD, including the development of comorbidities. Another important cause of comorbidities is smoking-induced oxidative stress. Smoking is a major cause of COPD and an important modifiable risk factor for the development of atherosclerosis [24,25]. A growing body of evidence supports the understanding that chronic inflammation and oxidative stress are important pathogenetic links for both COPD and atherosclerosis [26].

Currently, there is an ongoing debate regarding the understanding of comorbidities. Comorbidity was traditionally defined as the coexistence of two or more diseases linked by a single pathogenetic mechanism. It is well known that patients with COPD are prone to developing other diseases, such as coronary artery disease, lung cancer, osteoporosis, anemia, depression, and dysfunctional skeletal myopathy [27]. Previously, it was shown that only 28.4% of the patients with COPD had no comorbidities. However, the number of comorbidities depended on the severity of COPD, with cardiac comorbidities being more common in men [28]. There was also a significant association between an increased number of comorbidities and long-term mortality compared with patients without COPD comorbidities [29]. A high CCI score was associated with mortality [29].

ASCVDs play the most significant role in the comorbidities of COPD. ASCVDs are a global problem in modern medicine because of their significant contribution to the morbidity and mortality patterns of the population [30,31]. Comorbidities such as CAD and CHF were also shown to reduce the overall survival of patients with COPD. Simultaneously, the clinical heterogeneity of patients with COPD has an impact on survival rates. It was shown that patients with the chronic bronchitis phenotype have a lower survival rate for COPD exacerbations [32].

Numerous studies analyzed the relationship between COPD and cardiovascular disease, and these studies improved our understanding of these relationships. A key functional feature of COPD is the decline in lung function [33,34]. Spirometry data are used not only to diagnose the disease, but also to assess disease severity and prognosis. The association between lung function and 10-year risk of cardiovascular disease in healthy men and women in Italy was demonstrated [35]. Declining lung function is associated with an increased cardiovascular risk in the general population of China [36]. Patients with COPD with lower FEV1 and forced vital capacity (FVC) were shown to have a higher risk of CAD, cardiovascular diseases, and all-cause mortality [37]. FEV1 is a stronger predictor of mortality than FVC in patients with moderate COPD and increased risk of cardiovascular disease [38]. Another study showed that FVC was inversely associated with the 10-year risk of cardiovascular events, independent of the presence of metabolic syndrome and abdominal obesity in the general population without obstructive lung disease [35]. A growing body of evidence supports the understanding that it is not only the values of FEV1 measured over a period of time that matter, but also the rate of decline in FEV1 [33,39]. This indicator may reflect the individual characteristics of disease progression and may be related to comorbidities and prognosis. 

Declining lung function is closely related to the onset and severity of respiratory symptoms such as dyspnea [40]. Dyspnea is an important tool for assessing COPD progression [41]. Various multidimensional indices, including the BODE index, BODEX index, eBODE index, and CODEX index, were proposed to assess the course and prognosis of COPD, taking into account key clinical and functional characteristics of the disease, such as dyspnea severity, FEV1 scores, body mass index, exercise intolerance, exacerbation frequency, and comorbidity [42]. In the current study, it was shown that the comorbid course of COPD and ASCVD is characterized by higher values of multidimensional indices, which correspond to a more severe course and worse prognosis. 

Another important clinical characteristic of COPD is acute exacerbations [43,44]. COPD exacerbations lead to increased systemic inflammation, which increases the risk of cardiovascular disease development and complications. Subclinical atherosclerosis of the carotid arteries is more pronounced in patients with COPD exacerbation. Patients with COPD exacerbations have a higher risk of vascular ischemic events [45]. A large study conducted in the United States found that COPD exacerbations were associated with an increased incidence of acute cardiovascular events within 30 days and one year [46]. There is strong evidence that COPD is associated with an increased risk of myocardial infarction, with a higher risk of myocardial infarction during COPD exacerbation [47]. An increase in high-sensitivity cardiac troponin T levels was previously observed during exacerbations of COPD. High-troponin T levels are associated with increased mortality [48].

Thus, the data obtained in the present study highlight the importance of concomitant ASCVD in the course and prognosis of COPD. ASCVD is associated with decreased survival and should be considered when monitoring patient management.

### Study Limitations

It should be noted that this study had some limitations, such as its small sample size. In addition, only male patients participated in the study. This is due to the fact that men and women have different patterns of comorbidities. In addition, ASCVD progression depends on sex. Furthermore, the current study did not consider the specifics of disease pharmacotherapy and the specific medications prescribed in each patient group and subgroup. The strengths of our study include the long follow-up period and evaluation of disease progression at an intermediate time point of three years after the start of the study. The data from the current study can be used to design other studies to address these limitations and findings.

## 5. Conclusions

These results suggest that ASCVD influences the course of the disease and is the leading cause of mortality in patients with COPD. Therefore, the assessment of comorbidities is an important tool for assessing the course of COPD and should be widely used in clinical practice. The current study highlights the need for increased physician focus on early diagnosis of comorbid ASCVD in COPD. In addition, patient education programs aimed at early self-detection of comorbid ASCVD, including interpretation of symptoms, understanding of the natural history of COPD, and prognosis, could be potentially useful. Further studies with larger sample sizes are needed to confirm our findings and elucidate the potential mechanisms.

## Figures and Tables

**Table 1:** Clinical data of patients.

Characteristics	Data at the Time of Study Initiation
Age	60.02, 95% CI [58.68, 61.34]
Smoking	100% (170)
Pack-year index	37.72, 95% CI [36.41, 39.03]
Working in an air polluted environment	19.41% (33)
Body mass index (BMI)	26.6, 95% CI [26.13, 27.07]
FEV1 (% pred)	72.05, 95% CI [69.94, 74.17]
Dyspnea, mMRC	1.45, 95% CI [1.27, 1.62]

**Table 2:** Structure of comorbidities in patients.

Diseases	*n* = 170
Arterial hypertension, abs (%)	110 (64.7%)
Coronary artery disease (CAD), abs (%)	92 (54.11%)
Myocardial infarction (MI), abs (%)	11 (6.47%)
Chronic heart failure (CHF), abs (%)	73 (42.94%)
Acute cerebrovascular accident, abs (%)	10 (5.88%)
PAD, abs (%)	15 (8.82%)
Diabetes mellitus type 2, abs (%)	18 (10.58%)
Gastric and duodenal ulcer, abs (%)	17 (10%)
Liver and biliary tract diseases, abs (%)	16 (9.41%)
Diseases of the genitourinary system, abs (%)	19 (11.17%)

**Table 3:** Clinical and functional characteristics of COPD combined with ASCVD.

Parameter	Group with ASCVD (*n* = 92)	Group without ASCVD (*n* = 78)	*p*
Age	67.64, 95% CI [66.1, 69.18]	54.73, 95% CI [53.11, 56.36]	<0.001
GOLD 1 2 3	32 (34.78%) 50 (54.35%) 10 (10.87%)	21 (26.92%) 54 (69.23%) 3 (3.85%)	<0.001
FEV1 (% pred)	60.75, 95% CI [57.03, 64.47]	64.78, 95% CI [61.45, 68.11]	<0.001
Pack-year index	39.84, 95% CI [38.14, 41.53]	34.94, 95% CI [33.16, 36.71]	<0.001
Dyspnea, mMRC	1.89, 95% CI [1.62, 2.16]	1.17, 95% CI [0.93, 1.4]	<0.001
Duration of cough, years	18.71, 95% CI [17.2, 20.21]	12.06, 95% CI [11.09, 13.04]	<0.001
Duration of sputum production, years	14.11, 95% CI [12.77, 15.45]	8.85, 95% CI [7.72, 9.99]	<0.001
5-year survival rate	79 (85.86%)	74 (94.87%)	<0.001
10-year survival rate	29 (31.52%)	53 (67.94%)	<0.001
15-year survival rate	12 (13.04%)	39 (50%)	<0.001

**Table 4:** Clinical and functional characteristics of COPD combined with ASCVD at the second control point.

Parameter	Group with ASCVD (*n* = 92)	Group without ASCVD (*n* = 78)	*p*
BODE index	3.39, 95% CI [2.78, 4]	1.42, 95% CI [1.02, 1.83]	<0.001
eBODE index	4.67, 95% CI [3.99, 5.36]	2.49, 95% CI [2.06, 2.92]	<0.001
BODEX index	3.45, 95% CI [2.98, 3.91]	2.28, 95% CI [1.98, 2.58]	<0.001
CODEX index	4.45, 95% CI [3.94, 4.95]	2.41, 95% CI [2.08, 2.74]	<0.001
CCI	6.15, 95% CI [5.81, 6.49]	3.62, 95% CI [3.4, 3.83]	<0.001

**Table 5:** Results of Cox multiple regression analysis to assess risk factors for 15-year mortality in patients with COPD from COPD and ASCVD.

Factor	b	SE	Wald	*p*	Exp(b)	95% CI for Exp(b)
Rapid decline in FEV1	1.0082	0.2062	23.8973	<0.0001	2.7407	1.8294–4.1061
ASCVD history	0.6170	0.2096	8.6667	0.0032	1.8533	1.2290–2.7948
Exacerbations ≥ 2	0.9219	0.2088	19.4918	<0.0001	2.5142	1.6697–3.7857
Pack-years ≥ 30	0.7230	0.2417	8.9456	0.0028	2.0606	1.2830–3.3094

Notes: b—Cox regression coefficients; SE—standard error of the Cox regression coefficient; Wald—Wald’s χ2 tests the null hypothesis that the relative risk of death associated with a given variable is equal to one; significance—the level of significance achieved for the Wald’s χ2 criterion; Exp(b)—hazard ratio (HR), representing the increased or decreased risk of death at any time during the course of the disease associated with a single increase in its corresponding parameter, taking into account the effect of all other predictors; and 95% CI for Exp(b)—95% confidence interval for Exp(b).

## Data Availability

Data can be provided upon request to the author by e-mail.
